# Spatial analysis of the osteoarthritis microenvironment: techniques, insights, and applications

**DOI:** 10.1038/s41413-023-00304-6

**Published:** 2024-02-04

**Authors:** Xiwei Fan, Antonia Rujia Sun, Reuben S. E. Young, Isaac O. Afara, Brett R. Hamilton, Louis Jun Ye Ong, Ross Crawford, Indira Prasadam

**Affiliations:** 1 Centre for Biomedical Technologies, Queensland University of Technology, Brisbane, QLD Australia; 2 School of Mechanical, Medical & Process Engineering, Queensland University of Technology, Brisbane, QLD Australia; 3 Central Analytical Research Facility, Queensland University of Technology, Brisbane, QLD Australia; 4 Molecular Horizons, University of Wollongong, Wollongong, NSW Australia; 5 Department of Technical Physics, University of Eastern Finland, Kuopio, Finland; 6 School of Electrical Engineering and Computer Science, Faculty of Engineering, Architecture and Information Technology, University of Queensland, Brisbane, QLD Australia; 7 Centre for Microscopy and Microanalysis, University of Queensland, Brisbane, QLD Australia; 8 The Prince Charles Hospital, Brisbane, QLD Australia

**Keywords:** Pathogenesis, Diseases

## Abstract

Osteoarthritis (OA) is a debilitating degenerative disease affecting multiple joint tissues, including cartilage, bone, synovium, and adipose tissues. OA presents diverse clinical phenotypes and distinct molecular endotypes, including inflammatory, metabolic, mechanical, genetic, and synovial variants. Consequently, innovative technologies are needed to support the development of effective diagnostic and precision therapeutic approaches. Traditional analysis of bulk OA tissue extracts has limitations due to technical constraints, causing challenges in the differentiation between various physiological and pathological phenotypes in joint tissues. This issue has led to standardization difficulties and hindered the success of clinical trials. Gaining insights into the spatial variations of the cellular and molecular structures in OA tissues, encompassing DNA, RNA, metabolites, and proteins, as well as their chemical properties, elemental composition, and mechanical attributes, can contribute to a more comprehensive understanding of the disease subtypes. Spatially resolved biology enables biologists to investigate cells within the context of their tissue microenvironment, providing a more holistic view of cellular function. Recent advances in innovative spatial biology techniques now allow intact tissue sections to be examined using various -omics lenses, such as genomics, transcriptomics, proteomics, and metabolomics, with spatial data. This fusion of approaches provides researchers with critical insights into the molecular composition and functions of the cells and tissues at precise spatial coordinates. Furthermore, advanced imaging techniques, including high-resolution microscopy, hyperspectral imaging, and mass spectrometry imaging, enable the visualization and analysis of the spatial distribution of biomolecules, cells, and tissues. Linking these molecular imaging outputs to conventional tissue histology can facilitate a more comprehensive characterization of disease phenotypes. This review summarizes the recent advancements in the molecular imaging modalities and methodologies for in-depth spatial analysis. It explores their applications, challenges, and potential opportunities in the field of OA. Additionally, this review provides a perspective on the potential research directions for these contemporary approaches that can meet the requirements of clinical diagnoses and the establishment of therapeutic targets for OA.

## Introduction

Osteoarthritis (OA) is the most prevalent joint disorder, affecting an estimated 528 million people worldwide.^1,2^ It is currently defined by the Osteoarthritis Research Society International (OARSI) as a molecular malfunction accompanied by anatomic and physiologic degeneration, including cartilage degradation, bone remodeling, osteophyte production, and joint inflammation, and ultimately results in a loss of joint function.^3^ OA is the leading cause of activity restriction in people, as radiographic evidence of knee OA is found in approximately 30% of people aged 45 and older, with approximately 50% experiencing knee discomfort.^4,5^ OA is associated with a higher cost of living, lowered quality of life and shortened life expectancy in older adults worldwide.^6^ Although OA has become a severe health burden to society, no treatment options have emerged for slowed progression or cure of the disease because of the complexity of the disease, which involves the change of multiple cells, tissues, and organs.^7–9^ Current diagnosis and grading of OA often involve the combination of clinical symptoms, physical examination, and plain X-ray assessment (including observation of space narrowing, osteophytes, and bone-end deformity).^10^ However, X-rays provide limited information for nonmineralized tissues, such as cartilage, the tissue that is impacted in the disease.

Researchers have been continuously searching for early-stage biomarkers that may hint at disease onset, but unfortunately, no robust biomarkers have been found.^11^ There are two reasons for this phenomenon. First, OA is a multifactorial disease with various risk factors. Given the complexity of the pathogenesis and the seemingly unrelated patients’ symptoms during disease progression, one reason is potentially that OA disease metabolism is somewhat individual. Precision medicine, or personalized medicine, treats disease based on particular features, including an individual’s lifestyle or genetic vulnerability to disease and symptom progression. It is particularly applicable in OA due to its complicated and patient-diverse pathogenesis.^12,13^ Precision treatment heavily benefits from in-depth molecular-level information, mainly if molecular data can be related to specific diseased cell phenotypes or tissue structures. One way to access this information is through molecular-based imaging approaches, which provide information regarding the molecular level changes in disease because of their spatial format and can also associate these changes to histological features.

**Fig. 1:**
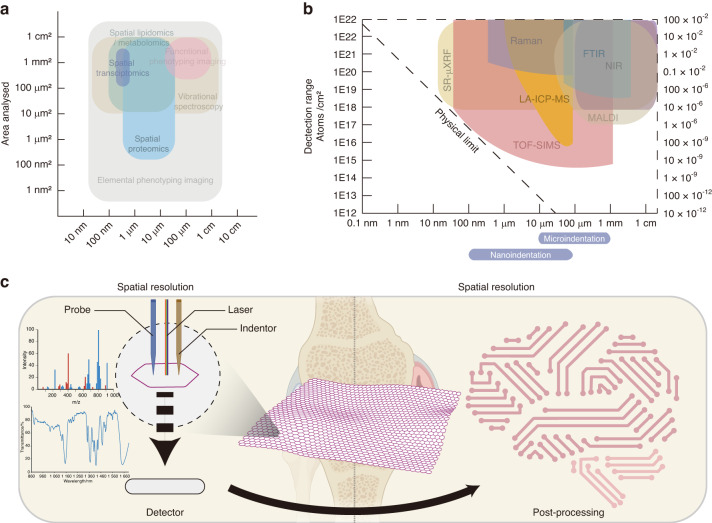
Magnitude and spatial resolution of the different instruments involved in deep spatial phenotyping. **a** Correlation map between the analyzed area and the spatial resolution of the state-of-the-art spatial phenotyping techniques used in the OA field. **b** Correlation map between the detection range and spatial resolution of the different instruments used for spatial phenotyping technologies. **c** Basic principle for deep spatial phenotyping in OA. This figure was created with Biorender.com

Second, the spatial architecture is very complex in the osteochondral unit. The osteochondral unit is a highly spatially organized structure containing uncalcified cartilage (superficial, transitional, and deep zone based on stratigraphy), tidemark, calcified cartilage, a cement line and a subchondral bone plate.^14^ The extracellular matrix (ECM) properties and molecular characteristics of chondrocytes and osteocytes differentiate the zonal architecture of cartilage, which changes in OA progression. Various imaging techniques have been developed to address this problem, including spectroscopic imaging,^15–17^ multiomics imaging,^18–20^ functional enzyme imaging,^21^ elemental imaging,^22^ and mechanical property imaging (Fig. 1a, b).^23^ Despite the differences in techniques, they all share the fundamental objective of generating spectral data within a single pixel in a localized region of the sample section. This spectral matrix is then integrated to produce the overall imaging information (Fig. 1c). In addition to imaging data acquisition, data processing, and postprocessing strategies, including artificial intelligence and deep learning techniques,^24,25^ have also been used in the biomedical area, providing great potential for risk assessment, molecular pathology, rapid diagnosis and precision medicine discovery for OA.

Combining many of the approaches used in previous OA research, deep spatial phenotyping, which refers to the use of advanced imaging technologies to quantify and analyze the morphological, functional and biochemical changes across a tissue matrix,^26^ is becoming increasingly crucial in OA understanding and potential treatment to overcome two previous technical difficulties in OA. Determining cell and tissue interactions at the spatial level, retaining the morphological landscape, and subgrouping the disease manifestations based on deep spatial organization would significantly enhance the understanding and avoid masking subtle changes from pathological tissues,^27^ thus facilitating future therapeutic applications.

Although spatial-type analyses can potentially benefit the OA field, few studies have been conducted to characterize and summarize the advancement of these techniques. In this study, the landscape of the modern imaging strategies that could be employed in OA-related research is introduced and clarifies the understanding of the OA phenotype at a spatial level in different scales.

## Different scales and different techniques for deep spatial phenotyping in OA

State-of-the-art techniques aid in providing a deeper understanding of OA at a molecular and spatial level, including distributions of DNA, RNA, proteins, metabolites, and elements; these usually require a combination of instruments and need to be selected based on the research purpose. These techniques, shown in Fig. 1b, have been used to gain an understanding of OA. Among them, the most promising advancements are based on vibrational spectroscopic imaging, multiomics imaging, functional enzyme imaging on tissues, elemental imaging, and mechanical property imaging. These techniques have varying degrees of spatial resolution, as outlined in Table 1. The summary of the findings from these imaging techniques at different scales is detailed below (Fig. 1).

**Table 1:** Comparison between the different advanced spatial phenotyping techniques for vibrational spectroscopic imaging

Imaging technique	Representative technique	Working Principle	Spatial Resolution	Advantages	Disadvantages	Metabolite detected	Application in OA
Vibrational spectroscopic imaging	NIR	Reflect C-H, N-H, and O-H stretching and bending overtones from 12 500 to 4 000 cm^−1^	100 μm^172^	Fast, accurate, nondestructive, labor-saving	Wideband, high spectral overlap, challenging to distinguish characteristic peak, hard to distinguish exact mass	GAG, PG^173^	High penetration but only full spectral signal of cartilage
	MIR/FTIR	Reflects molecular vibrational and rotational energy level transitions (4 000-400 cm^−1^)	10 μm^174^	Fast, accurate, and nondestructive; reflects the information of most organic matter	Low signal on bone, challenging to distinguish characteristic peak, hard to distinguish exact mass	PG,^48^ type II collagen,^50^ CS6^23^	Detects a multitude of components in the cartilage and can perform high-speed imaging
	Nano MIR/FTIR	Combining FTIR with s-SNOM. Based on AFM, an external light source illuminates a sharp tip, and the tip-scattered light (usually backscattered) is measured as a function of tip position	Up to 20 nm^42^	Fast, accurate, and nondestructive; reflects the information of most organic matter	Low signal on bone, difficult to distinguish characteristic peak, hard to distinguish exact mass	N/A	Detects a multitude of components in the cartilage, can perform high-speed imaging, and can detect subcellular changes
	Raman spectroscopy	Reflects the vibrational information between molecules based on the principle of Raman scattering	1 μm–250 nm^51^	Weak water signal, fast and straightforward, reflects the biological signal	The optical system and fluorescence interference can alter the Raman scattering region.	V_2_PO_4_^3-^, V_4_PO_4_^3-^, V_1_PO_4_^3-^, V_3_ PO_4_^3-^, V_1_CO_3_^2-^, Hydroxyproline, Amide I, Amide III, Saturated lipids, Unsaturated lipids, CH2 twist, GAG, PG, β(CH_2_) lipids, CH lipids, CH2 wag, β (CH_2_/ CH_3_) lipids, Tryptophan, Phenylalanine, Proline, Phenylalanine, Tyrosine^51^	Reflects physiological changes of OA and can reach subcellular level

*NIR* Near-infrared spectroscopy, *GAG* Glycosaminoglycan, *PG* Proteoglycan, *CS6* chondroitin 6-sulfate, *Nano MIR/FTIR* Nanoscale mid-infrared/Fourier transform infrared spectroscopy, *s-SNOM* Scattering-type scanning near-field optical microscopy, *AFM* Atomic force microscopy, *N/A* Not applicable

### Vibrational spectroscopic imaging

**Fig. 2:**
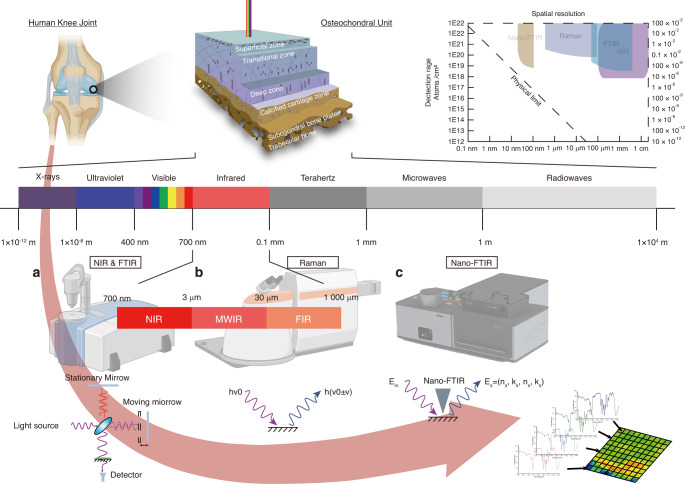
Pipeline of the different spatial phenotyping technologies in OA based on vibrational spectroscopic imaging. **a** NIR reflects C-H, N-H, and O–H stretching and bending overtones from 12 500 to 4 000 cm^−1^ with a resolution of 100 μm. It is fast, nondestructive, and labor-saving but has a wideband and overlap between different peaks in complex tissue, causing challenges in the differentiation of the characteristic peaks. MIR/FTIR reflects molecular vibrational and rotational energy level transitions (4 000–400 cm^−1^) with a resolution of 10 μm. It is fast, accurate, and nondestructive and reflects the information of most organic matter. However, it has a low signal on bone, causing challenges in the differentiation of the characteristic peaks and complexes for the determination of the exact mass. **b** Based on the principle of Raman scattering, Raman spectrometry imaging reflects the vibrational information between molecules with a resolution of 1 μm–250 nm. It is fast, accurate, and nondestructive, reflecting the information of most organic matter. However, the optical system and fluorescence interference can alter the Raman scattering region. **c** Nano-FTIR combines FTIR with scattering-type scanning near-field optical microscopy (s-SNOM). Based on AFM, an external light source illuminates a sharp tip, and the tip-scattered light (usually backscattered) is measured as a function of tip position. Its spatial resolution can reach 20 nm. The advantages and disadvantages are similar to FTIR. Advanced techniques enable the investigation of the microenvironment of cells and tissues at a subcellular level. NIR: Near infrared, MWIR: Middle wave infrared, FIR: Far infrared. This figure was created with BioRender.com

Vibration spectroscopy is a technique that enables material characterization based on light absorption by chemical bonds in molecules, including the chemical bonds between adsorbents and surfaces and those between adsorbed atoms. The resulting spectra provide information on the functional groups, chemical composition, and bonding environment of the materials and tissues. Spatial mapping is achieved by correlating the molecular bond changes and tissue-rich chemicals in the section. Vibrational spectroscopic imaging uses a diffraction grating to disperse light, and the light is then captured by charge-coupled devices similar to those used in digital cameras (Fig. 2). Due to the digital format, the extraction and analysis of one-dimensional spectra is easy, and high-sensitivity two-dimensional spectra are obtained. The resolved one-dimensional spectra can identify the specific functional groups, chemical species, and other molecular-level information.

In OA research, vibrational spectroscopic imaging has been used to investigate the chemical and molecular composition of the joint tissue, including cartilage, bone, synovial, and adipose tissues. Several vibrational spectroscopic imaging techniques are available and include near-infrared (NIR) imaging, mid-infrared (MIR)/Fourier transform infrared (FTIR) imaging, and Raman spectroscopic imaging. These techniques differ in the spectral region being probed, the types of information obtained, and the spatial resolution. To date, the integration of arthroscopy with various spectrometry techniques, such as NIR, has been used in preclinical studies.^28^ However, the techniques mentioned are not widely adopted in clinical practice for OA. By combining these techniques with other imaging modalities, such as histological analyses, researchers can better understand the molecular and cellular changes in OA.^29^ (Fig. 2). A detailed comparison of different vibrational spectroscopy results is shown in Table 1.

#### NIR imaging

In NIR spectroscopy, an unidentified substance is irradiated with broad-spectrum NIR light, and the light is then absorbed, transmitted, reflected, or scattered by the sample of interest. The NIR region of the electromagnetic spectrum spans from 780 to 2 500 nm (12 500–4 000 cm^−1^). Absorbance bands (overtones) in the NIR spectral range are primarily caused by bond vibrations between C-H, N-H, O-H, and S-H chemical bonds in the samples.^28^ These bands arise from the fundamental vibrations observed in the MIR spectrum region of 2 500–20 000 nm (4 000–500 cm^−1^),^30,31^ and represent the unique biomolecular composition of the sample. Based on the resulting spectrum, we can obtain essential information on a sample’s biochemical, molecular, and structural properties. Proteoglycan (PG) loss will change the C-H, N-H, O-H, and S-H bonds during OA progression. Therefore, specific algorithms can be used to capture these changes for the detection of early OA onset. NIR spectroscopy has various advantages, including rapid speed, nondestructive detection, and label-free evaluation. It is also a cost-effective optical analytical method compared to other spatial phenotyping technologies. Numerous organic substances have distinct NIR spectra, allowing NIR spectroscopy to be adapted as a useful analytical instrument. Due to its deep penetration into biological tissues, NIR spectroscopy is gaining attention in OA and disease diagnosis owing to a shift toward low-cost, portable, and less invasive disease identification methods.^32–35^

#### Histopathological OA detection and grading using NIR imaging on sections

NIR imaging has been suggested as a potential tool for investigating the etiology of OA on tissue sections by identifying biochemical changes related to glycosaminoglycan (GAG) and PG. Palukuru et al. found that the optimal NIR imaging thickness for cartilage tissue in its physiological state was 20–60 μm based on collagen and PG linear absorption band intensity variations at 1 336 and 856 cm^−1^.^36^ In their study, a partial least squares regression model was developed using NIR spectra in the range of 4 000–6 000 cm^−1^, and they successfully predicted MIR-derived bovine cartilage composition characteristics within 6% of the exact value. In addition to its utility for in vitro change detection, NIR has been integrated into spectroscopic methods for in situ detection.

#### Preclinical detection and grading of OA using NIR arthroscopy

Various studies have demonstrated the potential of NIR imaging for directly detecting early OA changes in in situ cartilage.^15,16^ Accurate classification and grading are advantageous for developing potential disease-modifying therapies in OA. Hofmann et al. examined the correlation of arthroscopy, magnetic resonance imaging (MRI), NIR, optical coherence tomography and spectroscopy with OA cartilage diagnosis. They discovered that the results obtained using NIR spectroscopy were in agreement with the Knee Injury and Osteoarthritis Outcome Score.^15^ Sarin et al. investigated the capacity of NIR spectroscopy for estimating cartilage thickness in vivo, as well as the PG concentration and collagen orientation angle.^16^ They found substantial diagnostic benefits using NIR spectroscopy for detecting disease-related changes in cartilage thickness and constituent structure using the neural network algorithm. The same group also investigated the capacity of NIR spectroscopy arthroscopically applied for estimating the biomechanical properties of cartilage in human cadaver knee joints and demonstrated the potential of the technique for clinical translation in arthroscopy. Afara et al. also reported that NIR spectroscopy could determine the composition of rat joint cartilage from the same tissue area.^37^ In particular, they demonstrated the capacity of NIR spectroscopy as a promising tool for monitoring OA progression in cartilage. They used multivariate spectral methods to classify (principal component analysis (PCA) and support vector machine(SVMs)) and predict (partial least squares regression (PLSR)) the Mankin scores and GAG content of the samples based on their NIR spectra, confirming NIR as a valuable tool for evaluating posttraumatic OA. Furthermore, AI-assisted techniques have been increasingly used to deeply understand the relationship between the chemical bond changes and OA alterations. A previous study demonstrated that combining NIR spectroscopy with machine learning algorithms enabled the classification of healthy and OA cartilage with high accuracy.^38^ The study showed that NIR spectroscopy coupled with machine learning could differentiate between the cartilage injuries resulting from anterior cruciate ligament transection (ACLT), nonoperatively operated control samples (by support vector machine), contralateral joints and nonoperated controls (by logistic regression) and the level of OA degradation (by deep neural networks). This progress builds a solid foundation for developing new approaches for arthroscopic evaluation incorporating the diagnosis of degenerated cartilage lesions and subchondral bone pathologies.

#### MIR/FTIR imaging

MIR and NIR have similar operating principles. However, the acquisition bands used by the two technologies are distinct. By illuminating samples with IR light, specific information on absorbed chemicals between 4 000 and 400 cm^−1^ can be obtained and examined.^39^ The distinct structure and arrangement can then be determined by the specific structure and arrangement of the sample. FTIR spectroscopy is an established method for identifying the micro- and macromolecular composition of biological materials and, like NIR, can be adapted into the form factor of a probe.^40^ Multiple advances have been developed for this classic instrument with novel imaging concepts by connecting robust optical imaging pipelines or AFM pipelines. FTIR microspectroscopy utilizes an FTIR spectrometer and a conventional optical microscope to identify the spatial distribution of diverse biochemical components and structures through chemical imaging.^41^ Furthermore, nano-FTIR was developed by combining the advantages of FTIR with scattering-type scanning near-field optical microscopy (s-SNOM).^42^ The working principle of nano-FTIR involves an external light source that illuminates a sharp tip using AFM, and the tip-scattered light (often backscattered) is measured as a function of tip location. After data acquisition, spectrum interpretation is at the spatial level. The sample’s structure and composition complexity cause challenges in the interpretation of the FTIR data; thus, additional analyses, including multivariate prediction models and partial least squares (PLS) regression, are needed to gain more insight into the target sample.^43^ To date, studies that have utilized FTIR imaging to investigate the osteochondral unit have focused exclusively on tissue sections rather than in vivo analysis. Further research is needed to evaluate the effectiveness of FTIR for early in situ OA detection.

#### Early detection and grading of OA combining MIR/FTIR imaging and algorithms

FTIR imaging has shown potential for the early detection of OA. FTIR imaging has the benefit over conventional imaging in that it can simultaneously identify both compositional and morphologic changes of OA tissue, thus enabling simultaneous diagnostic evaluation of biological tissues from the macro through micro to molecular levels. Combining the information from FTIR imaging with statistical and chemometric techniques, such as Fisher discriminant analysis (FDA) and PCA, provides a powerful approach for characterizing tissues by finding distinct features consistent with the sample groups. Using the methods above, Mao et al. collected FTIR images from normal and anterior cruciate ligament transection (ACLT) dog cartilage, performed PCA to find the principle features and then adapted them to the FDA model to determine OA classification.^44^ According to their findings, the classification succeeded with high accuracy for the training set (95.7%) and cross-validation (94.3%). Zhang et al. utilized FTIR imaging and partial least squares-discriminant analysis (PLS-DA) to distinguish between healthy and OA cartilage in dogs.^45^ They obtained a coefficient of determination of 0.95 between the actual and predicted values for the calibration set. Mao et al. further compared the results using FTIR imaging with PLS-DA and FTIR imaging with PCA–FDA in the normal and ACLT cartilage of dogs to validate which discriminant method was better and found that FDA methods were superior for a better understanding of OA pathophysiology.^46^ Using the combination of K-means clustering and PLSR, Oinas et al. also found that the collagen integrity and carbohydrates were closely related to disease classification.^43^ They further used the PLSR model to predict the OARSI grade and achieved high accuracy from cartilage superficial (94%) and deep zone (77%) structures.

#### Detecting biomarker changes by combining MIR/FTIR imaging and algorithms

Although MIR/FTIR detects unique signals from different materials (spectral fingerprint), similar or overlapping absorption bands (e.g., amide bands) shared by multiple substances decrease the accuracy in the determination of the changes in the constituent distribution and composition of tissues in OA. One good example is the overlapping IR spectra of PG and collagen of articular cartilage. However, by utilizing a second derivative spectral analysis, Rieppo et al. developed a feasible method for analyzing articular cartilage composition using FTIR to improve its resolution for individual constituents.^47^ Moreover, multivariate regression models capable of predicting cartilage PG content have been developed to increase further accuracy and sensitivity^48^ obtained by Rieppo et al. The FTIR imaging technique was used to quantitatively determine the zonal profiles of the proteoglycan concentration and the distribution of collagen fibrils in healthy and osteoarthritic articular cartilage of experimental models.^49^ They found reduced PG in the OA group compared with the control group. However, no significant changes were found in collagen, and they concluded that early-stage OA had minimal effect on collagen content. FTIR imaging was used by David-Vaudey et al. to examine human cartilage after total knee replacement. Type-II collagen and chondroitin 6-sulfate (CS6) were detected using quantitative partial least squares analysis and Euclidean distance mapping after dividing the sample into healthy and normal groups and comparing them based on stratigraphy (superficial, middle, and deep zones).^50^ Furthermore, they found a decrease in type-II collagen, PG, and CS6, confirming the feasibility of FTIR imaging for assessing tissue pathology.

#### Raman spectroscopic imaging

Raman scattering (Raman effect) is an inelastic scattering phenomenon of photons, which refers to a change in the frequency of light waves after being scattered. The Raman effect is observed by irradiating a sample with a high-intensity laser and using a spectrometer to measure the resulting scattered light. The difference in energy between the incident radiation and the scattered light is known as the Raman shift and is measured in wavenumber (cm^−1^). Raman shifts at higher wavelengths (nm) are known as Stokes scattering, and Raman shifts at lower wavelengths (nm) are known as antistokes scattering. While Raman and IR both result from bond vibrations, they differ in that IR results from light absorption at specific wavelengths by bonds, whereas Raman relies upon changes in bond polarizability where light can be scattered (the Raman shift). By utilizing this working principle, Raman spectroscopy has been widely used in the OA field because the chemical bonds and symmetric molecules have vibrations that produce unique spectral information, providing essential characteristics for molecule identification. Raman spectroscopy is easy to use and is not affected by water in biological tissues, and it is also capable of high spatial resolution (1 μm–250 nm),^51^ which can be extremely valuable for the early detection of OA at the subcellular level. To date, research using Raman imaging to examine the osteochondral unit has been limited to studying tissue samples rather than live, in-body assessments. Additional studies are needed to optimize Raman spectroscopy for the identification of early OA detection in clinics.

#### Detecting biomarker changes using Raman spectroscopic imaging

OA is characterized by subtle molecular changes to the osteochondral unit at the early stage and can be investigated using Raman spectroscopy.^52^ Gamsjaeger et al. first used Raman spectroscopic imaging to validate the feasibility of Raman spectroscopy, and they successfully found various biomarkers, including PG, amide I, amide III, PG/amide III, PG/amide I, and V_2_PO_4_/amide III, across the osteochondral interface obtained from the human femoral head.^17^ Bergholt et al. also characterized zonal differences in articular cartilage using Raman spectroscopic imaging to identify collagen, GAG, water distribution and collagen fiber orientation.^53^ Following their study, Albro et al. further quantified the depth-dependent heterogeneity of both native and engineered cartilage.^54^ Based on that, Gupta et al. graded human samples into healthy and OA samples and used Raman spectroscopic imaging to visualize and evaluate the sample using multivariate cluster analyses. They found various compositional differences in V_4_PO_4_^3−^/amide III, V_4_PO_4_^3−^/(hydroxyproline + proline), V_1_CO_3_^2−^/ V_4_PO_4_^3−^, V_1_CO_3_^2−^/amide I, proteoglycan/amide III, saturated lipids/amide III and unsaturated lipids/amide III.^51^ With the abovementioned Raman spectrometry imaging in vitro, researchers have also attempted to combine Raman with existing arthroscopy instruments. Gaifilina et al. measured sGAG at arthroscopy and found significant waveband changes, which reflected early OA prior to lesion formation.^55^ Their study provided a theoretical foundation for future research on probe-based Raman spectroscopy for the early detection of OA. Based on the potential of Raman spectroscopy for detecting various components in bone and cartilage samples with minimal interference from water, it has been adapted for the early detection and grading of OA. Kerns assessed the capacity of Raman spectroscopic imaging for the early detection of OA^56^ and found that lesions existed beneath the unaffected knee compartments; these were more vulnerable to OA onset. Similarly, Gupta et al. found increased mineralization activity in early OA but decreased mineralization activity in advanced OA, providing novel evidence of tissue-specific changes during OA progression.^51^ In addition to its application in the analysis of bone and cartilage, Raman spectroscopy has been employed for OA stratification using synovial fluid^57^ and synovium.^58^ However, acquiring in situ spatial imaging comparable to the resolution of MRI or computed tomography remains challenging with Raman spectroscopy.

### Spatial multiomics imaging

Molecular interactions exist within the tissue and cell, among various biological layers from the genome and epigenome to the transcriptome, proteome, and metabolome.^59^ Multiomics imaging has proven to be a key component of the spatial-temporal analysis at the systematic level^60^ and is especially suitable for multifactorial diseases such as OA. Despite the considerable success of multiomics imaging for disease and treatment evaluation in other diseases,^59,61^ spatial multiomics imaging in the OA field has not been performed. To date, multiomics imaging techniques have been widely used for preclinical studies in OA etiology, but none have been used in clinical practice for OA. A thorough examination of recent progress provides insights into accomplished work and areas that still require further research is provided here. Detailed comparisons of the current techniques are shown in Table 2.

**Table 2:** Comparison between the different advanced spatial phenotyping techniques for multiomics imaging

Imaging technique	Representative technique	Working Principle	Spatial Resolution	Advantages	Disadvantages	Metabolite detected	Application in OA
Spatial transcriptomics	10X Visium	Integrate oligos into the slides and combine them with tissue	Up to 200 nm	Detects RNA signal spatially, with high accuracy and sensitivity	Expensive, compatibility issue in bone and cartilage sample	Multiple RNA expressed spatially in the synovium^19,71^	Reflects the spatial RNA expression in the OA tissue
Spatial proteomics	MALDI-TOF	Use certain protein enzymes to dissect the protein sequencing into peptide segments. The substrate can ionize the sample. Then, MS imaging instruments can extract mass for the mapping	20 μm^175^	Detects peptide signal spatially, mid to high sensitivity	Substrate needed, hard to interpret, specific peptide signals point to various proteins, need further validation	ACAN, BGN, CILP-1, COMP, COL-2A-1, DCN, FMOD, FN, Prolargin, ELY-S,^79^ Fibronectin,^59,60^ N-glycans,^79–81^ Calgranulins, Defensins, Thymosins,^176^ ACTA, HBA-2, HBB,^20^	Reflects the spatial protein expression in the OA tissue
	DESI imaging	The same as MALDI-TOF but does not need to use substrate to ionize the sample in advance	50 μm^175^	Detects peptide signal spatially, with no need for substrate, mid-sensitivity	Specific peptide signals point to various proteins that need further validation	N/A	Reflects the protein spatial expression in the OA tissue
	DSP	A special antibody-UV linker-encoded oligo complex was stained, and then specific regions of interest were exposed to UV laser to cleave oligo for sequencing and digital counting.	1 μm^83^	Simultaneously, detects RNA and protein in the region of interest, suitable for fixed tissue.	There is a need for transparent sections. No direct image but ROI is generated through this technique	N/A	Reflects the protein and RNA expression in the small region of interest in the OA tissue
	IMC	Combines laser ablation and time-of-flight cytometry for the identification of metal-tagged antibody-labeled targets.	1 μm^177^	High accuracy and sensitivity, can detect up to 40 kinds of protein in one batch	Relevant antibody needed, difficult to explore unknown protein	N/A	Accurately reflect the spatial expression of targeted proteins in the OA tissue.
	MIBI	Combines ion beam and SIMS for the identification of metal-tagged antibody-labeled targets.	350 nm–1 μm^178^	High accuracy and sensitivity, can detect up to 54 kinds of protein in one batch	Relevant antibody needed, difficult to explore unknown protein	N/A	Accurately reflect the spatial expression of targeted proteins in the OA tissue.
Spatial lipidomics	MALDI-TOF	Use a specific substrate to ionize the sample. Then, MS imaging instruments can extract ionized lipids for mapping	20 μm^175^	Detects lipid signal spatially, mid to high sensitivity and accuracy	Specific mass/charge ratio points to various substances that need further validation	SM (36:1), PC (32:0), PC (36:1) and PC (36:2), PI, PE^179^	Reflects the lipids’ spatial expression in the OA tissue.
	TOF-SIMS	Dislodges chemical species on a material’s surface using a focused, pulsed particle beam and detects the time of fight of the produced dissociated ions for lipid detection.	0.1 μm	High resolution, high accuracy and sensitivity	Only obtain the functional group of the lipid. It is difficult to know the exact lipid species and isotype	vitamin D-3, prostaglandins, glycerolipids, DAG, C18:0, C18:1, C18:2, C16:0, C16:1^95^	Reflects the spatial lipids functional group and lipid group with low molecular weight in the OA tissue.
Spatial metabolomics	MALDI-TOF	Use a specific substrate to ionize the sample. Then, MS imaging instruments can extract ionized metabolites for the mapping.	20 μm^175^	Detects metabolites spatially, mid to high sensitivity and accuracy	Specific mass/charge ratio points to various substances that need further validation	ATP, ADP, AMP, UDP, UMP, GDP, GTP, GMP and ADP-ribose^180^	Reflects the metabolites’ spatial expression in the OA tissue.
	TOF-SIMS	Dislodges chemical species on a material’s surface using a focused, pulsed particle beam and detects the time of fight of the produced dissociated ions for metabolite detection.	0.1 μm	High resolution, high accuracy and sensitivity	Only get the functional group of the lipid. It is hard to know the exact metabolite species with a large mass/charge ratio	N/A	Reflects the spatial expression of metabolites with small molecular weight in the OA tissue.
Functional phenotyping imaging	MALDI-TOF	Use specific substrates for the enzyme to digest. Then, MS imaging instruments can extract ionized product signals for mapping.	20 μm	Detects substrate and products spatially with high sensitivity and accuracy	The targeted experiment needs to validate the existence of the enzyme first.	PLA2^104^	Reflects the spatial expression of enzyme activity in the OA tissue.

*RNA* Ribonucleic acid, *OA* Osteoarthritis, *MALDI-TOF* Matrix-assisted laser desorption ionization-time of flight mass spectrometry, *ACAN* Aggrecan core protein, *BGN* Biglycan, *CILP-1* Cartilage intermediate layer protein 1, *COMP* Cartilage oligomeric matrix protein, *COL-2A-1* Collagen alpha-1(II) chain, *DCN* Decorin, *FMOD* Fibromodulin, *FN* Fibronectin, *ELY-S* Protein embryonic large molecule derived from yolk sac, *ACTA* Actin aortic smooth muscle, *HBA-2* Hemoglobin subunit-alpha-2, *HBB* Hemoglobin subunit beta, *DESI* Desorption electrospray ionization, *DSP* Digital spatial profiler, *ROI* Region of interest, *IMC* Imaging mass cytometry, *MIBI* Multiplexed ion beam imaging, *PC* Posphatidylcholines, *SM* Shingomyelin, *PE* Phosphatidylethanolamines, *PI* Posphatidylinositol, *DAG* Diacylglycerols, *TOF-SIMS* Time-of-flight secondary ion mass spectrometry, *ATP* Adenosine triphosphate, *ADP* Adenosine diphosphate, *AMP* Adenosine monophosphate, *UDP* Uridine diphosphate, *UMP* Uridine monophosphate, *GDP* Guanosine-5'-diphosphate, *GTP* Guanosine triphosphate, *GMP* Guanosine monophosphate, *ADP-ribose* Adenosine diphosphate ribose, *PLA2* Phospholipase A2, *N/A* Not applicable

#### Spatial transcriptomics

Spatial transcriptomics is a subfield that focuses on mapping the spatial distribution of RNA transcripts within cells, tissues, and organisms.^62,63^ Understanding gene and protein expression in the tissue microenvironment is critical for understanding the pathophysiology of complex diseases such as OA. Although decades of genome-wide research have yielded a wealth of knowledge regarding the gene regulatory networks specific to different cell types, comprehending the interactions between cells and their environment outside the tissue is still limited. The increase in technological advances in recent years has enabled system-level characterization of cellular heterogeneity and spatial organization of tissues/organs. The rapid development of single-cell RNA-sequencing technology (scRNA-seq) applications, which has provided the ability to profile and compare the gene expression patterns of many individual cells within a tissue/organism, is perhaps the most notable. However, creating a single-cell suspension through mechanical and enzymatic dissociation steps is crucial in the experimental procedure and inadvertently destroys the original tissue architecture. Several technologies for transcriptomic profiling and spatial information preservation have been recently developed. These spatial-level genomic technologies have been acknowledged to revolutionize biological research in the future.^18,19^ The basic concept of spatial transcriptomics is integrating oligos onto the slides, followed by applying the tissue section, which is permeabilized to release RNA bound to adjacent capture probes, and thereby captures the gene expression information. Complementary DNA (cDNA) is synthesized from the captured RNA and sequencing library. Spatial transcriptomics has been used for various tissues in OA. Single-cell transcriptomics has shown that various subtypes of chondrocytes exist in OA cartilage.^64^ Probabilistic approaches could combine the existing single-cell data with spatial RNA-seq data to investigate the geographic correlations between previously recognized cell types and states.^65^ This approach enables investigation of the geographical distribution of cell types shown by single-cell sequencing and provides information on the relative accessibility of these cells to synovial fluid mediators or any intra-articular therapies. Another cost-efficient technique includes digital spatial profiling, which surrounds an area within the tissue for gene/protein expression analysis.^66,67^

**Fig. 3:**
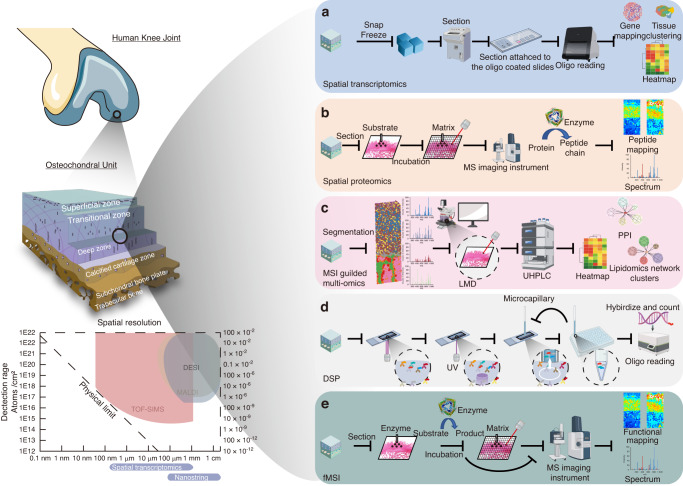
Pipelines of the different multiomics spatial phenotyping technologies used in the OA field. **a** Spatial transcriptomics is a cutting-edge technology that merges oligonucleotides with slides and combines them with tissue samples for detection. This method enables the detection of RNA signals with high accuracy, sensitivity, and a resolution of 200 nanometers. However, this technology is relatively costly and has potentially compatibility challenges when applied to bone and cartilage specimens. **b** Spatial proteomics uses certain protein enzymes to dissect the protein sequence into peptide segments. The sample is then ionized using different techniques, including matrix-assisted laser desorption/ionization (MALDI) and desorption electrospray ionization (DESI); finally, the laser is used to extract the mass for mapping with a resolution up to 20 μm. It spatially detects peptide signals, with mid to high sensitivity. However, the data is difficult to interpret. Specific peptide signals point to various proteins, and they need further validation. **c** Mass spectrometry imaging (MSI)-guided multiomics combines MSI and laser microdissection microscopy for separation, cross-validation and analysis. It can reach high accuracy and sensitivity but requires an optimized section-transferring system. **d** Digital spatial profiler (DSP) employs barcoded fluorescent markers for RNA and protein detection. It is easy to use with multiple genes and protein detection capability available, although it shows only partial information in the gene clusters without mapping. **e** Functional mass spectrometry imaging (fMSI) uses specific substrates for digesting the enzyme. Then, MSI instruments can extract ionized product signals for mapping with a resolution of up to 20 μm, confirming high sensitivity and accuracy when spatially detecting substrates and products. However, researchers need to first validate the existence of the enzyme. This figure was created with BioRender.com

To date, spatial transcriptomics has evolved into a spatiotemporal transcriptome that dynamically traces gene expression in the whole genome.^68^ The nanoball array enables the spatial resolution to be approximately 200 nm, thus providing sufficient in-depth subcellular information. Spatial RNA sequencing has been described as a future sequencing approach that allows visualization and quantitative analysis of transcriptomics from the entire histological tissue sections while preserving important tissue architectures and features.^69^ The recent development of spatial transcriptomics, such as commercially available 10X Visium and NanoString GeoMx technologies, is applied to further reveal unified spatiotemporal changes and molecular fingerprints in the cartilage and synovium.^70,71^ Initial research examining the synovium with the application of spatial transcriptomics focused on a comparative analysis between individuals diagnosed with rheumatoid arthritis (RA) and those with spondyloarthritis (SpA). The findings indicated a higher prevalence of central memory T cells in the RA cohort, whereas effector memory T cells were more prevalent in the SpA group.^19^ Following this, studies on the morphological tissue features and cell type-specific localization patterns in the human rheumatoid arthritis synovium by applying spatial transcriptomics to uncover cell-to-cell interactions at the site of inflammation provide technical models for gathering new knowledge on the molecular profiles of the synovial tissue and identifying which cells are involved in the pathogenesis of OA.^19,71^ Subsequent research has integrated single-cell RNA sequencing (scRNA-seq) with spatial transcriptomics to reveal a marked elevation in the population of pericytes and endothelial cells within osteoarthritic tissue relative to the synovium affected by femoroacetabular impingement.^72^ In a more recent study utilizing 10X Genomics, Kulzhanova et al. identified three distinct zonal chondrocyte populations in the knee cartilage of the E18.5 mouse embryo, ranging from the superficial zone to the deep zone. They found the potential hub genes governing chondrogenesis in vivo.^70^ This technique makes it possible to investigate the spatial distribution of various chondrocyte phenotypes, map the spatiotemporal transcriptomic dynamics, alter molecular mechanisms regulating chondrocyte activities concerning cartilage degradation during the development of OA, and provide insights into possible therapeutic targets. Nevertheless, some challenges remain for spatial transcriptomics in OA. Until now, 10X Genomics has not been applied to bone and cartilage samples due to sample preparation compatibility issues. Moreover, the resolution of 10X transcriptomics is higher than the size of the cells. Therefore, the osteochondral unit change at a spatial level is difficult to investigate. The more in-depth analyses rely on subcellular investigation using a fresh frozen sample pipeline, including Kawamoto’s film methods and other tape-assisted systems (Fig. 3).^73^

### Spatial proteomics

Spatial proteomics refers to a type of proteomics that focuses on analyzing the spatial distribution of proteins within cells, tissues, and organisms.^18,74^ Canonical proteomics and western blotting are reliable and reproducible methods for qualitative and quantitative protein analysis extracted from tissue samples. However, these methods are typically bulk analyses, and the extraction aspect removes the spatial information from the analysis. The bulk analysis does not involve the distribution of analytes throughout the tissue, which is an essential consideration in any pathological tissue analysis. The lack of spatial information hampers developing a fundamental understanding of OA at the cellular or tissue pathology level from the protein perspective. Owing to this, new spatial phenotyping techniques have thrived in recent years and made significant advancements in the OA field. The development of the application of state-of-the-art spatial proteomics techniques in the OA field will be described.

#### Untargeted spatially resolved exploration by mass spectrometry imaging (MSI) in OA

Due to its capability to resolve protein levels in situ, spatial proteomics has been gaining popularity in the OA field; here, MSI is one of the outperforming techniques that provides spatial information. Typically, to visualize proteins with MSI, the tissue section is processed using a proteolytic enzyme to elaborate peptides from the proteins in the tissue section. Observation of specific peptides during MSI then shows the presence of the protein. MSI has limited the ability to both detect and identify intact proteins. However, detection and identification has become much more achievable when enzymatically analyzing the elaborated peptides due to the improved sensitivity, mass accuracy and mass resolving power at lower *m/z* values where mass spectrometers observe peptides. MSI allows the visualization of tissues pixel by pixel, where the molecules in each pixel are ionized and analyzed by a mass spectrometer. MSI identifies peptides by comparison of the observed and theoretically accurate mass of the peptide sequence. The sequence can be confirmed using MS fragmentation approaches such as LC‒MS and occasionally using direct off-tissue tandem MS. However, off-tissue tandem MS requires a good signal from the precursor to record the tandem MS spectrum. The identification of a protein can often be confirmed by the observation of multiple peptides emanating from enzymatic digestion in MSI analysis. This approach is reminiscent of peptide mass fingerprinting, which is commonly used to identify one-dimensional and two-dimensional gel bands in proteomic analysis. There are a variety of analyte ionization approaches used for MSI, including matrix-assisted desorption ionization (MALDI), desorption electrospray ionization (DESI), laser-assisted electrospray ionization (LA-ESI), liquid extraction surface analysis (LESA), and focused ion beams. Various mass analyzers, including axial time-of-flight (TOF), are very fast; however, the tissue surface topography can affect the mass resolution, and the laser power significantly influences the mass resolution. High mass resolving Fourier transform analyzers include Orbitrap and Fourier Transform Ion Cyclotron Resonance (FT-ICR) instruments, which provided a very high mass resolution; however, the duty cycle for these analyzers is long, causing significantly longer acquisition run times than TOF analyzers. Recently, hybrid quadrupole orthogonally accelerated time-of-flight (oaTOF) mass analyzers have been increasingly used for MSI. The oaTOF instruments provide improved mass accuracy and mass resolution compared to the axial TOF instruments and increased acquisition speed compared to the FT mass analyzers. The current state-of-the-art with respect to oaTOF instruments typically provides correct mass measurements to the second decimal place; this confers reasonable confidence in the molecular formula of the observed species. Several enzymes can be used for catalyzing the tissue for proteomic analysis, including trypsin, chymotrypsin, Elastase, Pepsin, N-glycosidase F (PGNase F), collagenase^75^ and others.^76^ Laser microdissection microscopy (LMD)-guided MSI is another technique that potentially detects tissue level changes by carefully dissecting the region of interest. LMD can extract tissue from a more delicate area, relying on the laser intensity and diameter. However, histology alone does not always provide accurate natural demarcation, especially for cartilage with complex zonal architecture; thus, LMD samples may require more than just optical inspection of the tissue section when selecting areas of interest. In this case, MSI and its unique PCA clustering can benefit from an artificial intelligence bioinformatics pipeline providing a landscape of the entire cartilage layer. Combining MSI acquired with an oaTOF instrument and LC‒MS proteomic analysis of small regions of interest collected using LMD allows for improved confidence in protein identification during MSI. Reliance on accurate mass measurement alone is not always sufficient to identify a peptide; for example, one observed *m/z* may correspond to more than one peptide sequence.

Using MALDI-MSI, Cillero-Pastor et al. discovered a specific protein profile discriminating control from synovial tissues of OA patients in a spatially resolved manner for the first time.^20^ Fibronectin peptides were preferentially upregulated in hypertrophic and inflammatory regions of the OA synovium, contributing to tissue remodeling, inflammation and cell death.^77,78^ The spectra also showed the same candidates in the OA cartilage, demonstrating that fibronectin was a common feature in OA tissues. Recent studies using MALDI-MSI analysis have been conducted to identify and localize proteins, tryptic peptides and lipids from OA and healthy articular cartilage. Apart from fibronectin, the altered structure of N-glycans, which are protein posttranslational modifications, has been reported in the deep layer of OA cartilage, and high mannose N-glycans showed distinct spatial distribution throughout OA tissues.^79–81^ The recent development of transmission mode MSI has enabled subcellular analysis mapping, which is expected to deeply improve our knowledge of OA and the biomolecules that are important during its onset and progression.^82^

#### Targeted multiplexed imaging techniques for spatial proteomics in OA

In addition to various MSI techniques, additional new multiplexed imaging techniques are emerging with enormous potential in spatial proteomic analysis of OA samples; the most notable these techniques are digital spatial profiler (DSP),^83^ Imaging Cycler (toponomix),^84^ multiplexed ion beam imaging (MIBI),^85,86^ and imaging mass cytometry (IMC or cyTOF/Hyperion).^87^ Moreover, the integration of these with prior MSI investigations in OA provides targeted validation that is anticipated to confirm these alterations and show its applicability for future applications in the field.

DSP is a high-throughput imaging method that enables the simultaneous analysis of many protein markers in a single tissue sample.^83^ The working principle of DSP is to stain sections with a special antibody-UV linker-encoded oligo complex and then cleave oligo using a UV laser for downward sequencing and digital counting. DSP is able to detect of hundreds of protein markers in a single tissue sample, provided a comprehensive perspective of the joint’s protein landscape. DSP requires a transparent section and does not provide an optical image of the section; instead, a predefined region of interest (ROI) is generated and analyzed. Presently, this technique is mainly utilized in cancer research.^88,89^ Nevertheless, its potential as a valuable instrument in OA research provides a promising avenue for future exploration.

The Imaging Cycler is a microscope-based technology that uses fluorescence protein tagging.^84^ The approach essentially relies upon robotically controlled incubation-imaging-bleaching cycles. This allows for multiple numbers of proteins to be imaged from the same tissue, with a correlation of typically 40-100 proteins per section. Using incubation-imaging-bleaching cycling means that the cycling achieves multiplexing rather than requiring multiple fluorescent tags. The spatial resolution can be as low as 40 nm.^90^

MIBI is a secondary ion mass spectrometry (SIMS) approach that uses a focused ion beam (FIB) to generate charged ions from the surface of the tissue.^85,86^ MIBI uses tissue sections exposed to antibodies chelated by various metal ions. The metal ions chelated to the antibodies are observed as secondary ions due to the FIB hitting the surface of the tissue. In the MIBI, the observation of a specific metal ion shows where the antibody tagged with that metal ion is present in the tissue. Essentially, multiplexing for multiple proteins is achieved by having antibodies to proteins of interest be chelated with different transition metal isotopes. The spatial resolution can be as low as 280 nm, and at present, the multiplexing is approximately up to 40 proteins. The limitation of multiplexing is how many different transitional metal isotopes can be used to differentially label the different antibodies. Imaging mass cytometry (IMC) is an approach that also uses mass spectrometry; in this case, it uses a laser ablation inductively coupled plasma‒mass spectrometry (LA-ICP) instrument, which is typically used for elemental analysis.^87^ The IMC instrument without laser ablation is used for mass cytometry and is known as the CyTOF, and the LA-ICP‒MS instrument is known as the Hyperion. The IMC instrument has a time-of-flight (TOF) mass spectrometer that differentiates molecules based on smaller ions traveling faster to the detector. The IMC approach is quite similar to the MIBI in that transition metal-labeled antibodies are used to locate the proteins, with the detected transition metal ion showing the protein’s location. In the Hyperion instrument, the laser ablates the tissue, and this material is transferred to the inductively coupled plasma, where it is atomized by the argon plasma, and then the TOF measures the generated ions. The Hyperion can easily multiplex because each spectrum collected spans a mass range; thus, the major limitation of the ability to multiplex is the number of transition isotopes available for use. Typically, IMC can multiplex to approximately 50 proteins and can have a spatial resolution of 1 μm. Both IMC and MIBI will likely play a crucial role in the future spatial proteomic analysis of OA samples. In summary, the aforementioned advanced technologies are gaining attention in the field of OA, and they will undoubtedly lead to innovative and effective treatments for OA.

### Spatial lipidomics

Lipidomics refers to the study of the complete set of lipids in an organism or cell and their functions, and spatial lipidomics is used to understand where these lipids are located within the cell or tissue and how this spatial information can be used to gain insight into the cellular processes and diseases.^91,92^ In joint homeostasis, lipids are mainly located on the joint surface and bone marrow, providing five functions reducing friction, inducing chondrogenesis, chondrocyte viability, matrix synthesis, and transducing cell signaling.^93^ A recent study indicated that the scarcity of lipids could induce chondrogenesis in cartilage,^94^ highlighting the importance of lipids in joint homeostasis and their spatial changes during OA progression.

Despite numerous efforts, the exact changes at the spatial level during the progression of osteoarthritis need to be fully investigated. Cillero-Pastor et al. first reported human OA cartilage lipid profiling with SIMS-TOF in 2012.^95^ Their study reported spatial distribution differences regarding vitamin D-3, prostaglandins, glycerolipids, diacylglycerol (DAG), C18:0, C18:1, C18:2, C16:0, and C16:1. Rocha et al. recently discovered that OA patients had distinct lipidomics patterns compared to rheumatoid arthritis (RA) and psoriatic arthritis (PsA) patients. Compared to control tissues, OA synovium had higher levels of phosphatidylcholines, fatty acids, and lysophosphatidic acids but lower levels of lysophosphatidylcholine (LPC). The geographical distribution of specific glycerophospholipids was similarly associated with hypertrophic, inflammatory, or vascularized synovial regions. Compared to other forms of inflammatory arthritis, OA tissue contained fewer phosphatidylethanolamine-based plasmalogens.^96^ Using MALDI-TOF, Eveque-Mourroux et al. compared OA cartilage with or without type 2 diabetes mellitus (T2DM).^97^ Their results demonstrated that phospholipid (PC) and sphingomyelin (SM) species were characteristic of the surface layers and were more prevalent in the OA/T2DM– group, whereas lysolipid species were more indicative of the deep layers and were more prevalent in the OA/T2DM+ group. In addition to the osteochondral unit, fat pad changes have also been studied in OA progression. In one study, the application of MALDI-MSI revealed a significant distinction among patients, where those that were affected by OA exhibited heightened concentrations of phospholipids compared to their counterparts with cartilage defects. Moreover, the study revealed a specific emphasis on the prevalence of ether-linked phosphatidylethanolamines (PE O-s), including arachidonic acid, which demonstrated an increase in individuals with OA as opposed to those presenting with cartilage defects. These findings not only clarify the lipid composition variations but also emphasize their potential significance in distinguishing between OA and other conditions related to cartilage.^98^ Although few advancements have been made, the exact lipid classes need be cited with caution since different lipid species share the same precursor mass for lipid ID, where different lipids can have the same molecular formula; therefore, follow-up structure analysis is often needed. Nevertheless, spatial lipidomics still provide an unprecedented vision for the lipid changes in OA at the spatial level.

### Spatial metabolomics

In addition to RNA, protein and lipids, multiple metabolites play a role in maintaining tissue homeostasis. A famous example is N-glycans; its measurement is crucial in OA since these analytes are closely related to disease progression.^99^ MALDI-MSI has been used to spatially determine N-glycome in the cartilage and subchondral bone of individuals with knee OA.^81^ MALDI-MSI was used to identify approximately 40 N-glycan structures in cartilage and subchondral bone proteins. High-mannose N-glycans were spatially distributed differently in the OA tissues. Notably, (Man)3 + (Man)3 (GlcNAc)2 was found to be higher in cartilage than in subchondral bone. In addition, (NeuAc)2 (Hex)2 (HexNAc)2+ (Man)3 (GlcNAc)2 profiles were used to differentiate between OA patients with distinct bone marrow lesion phases. In conjunction with MSI, PNGase F and trypsin can increase the number of protein identifications while preserving the spatial information. Recent literature describes MALDI-MSI analysis of N-linked glycans and proteolytic peptides from the same tissue segment. Researchers successfully digested slices of cancerous tissue with PNGase and trypsin enzymes.^100^ In addition to previous studies, few studies have been published on metabolite changes in OA at a spatial level. The validity issue for identifying multiples by only matching the molecular weight or the m/z ratio needs to be addressed. More research needs to be performed to provide better validation at the current stage.

### Functional enzyme imaging of tissues

Multiple enzymes play vital roles in the degeneration of the osteochondral unit during OA progression,^101^ particularly the increase in matrix metalloproteinases (MMPs) and the decrease in proteinase inhibitors. Commonly acknowledged proteinases include collagenase, stromelysins, and gelatinases, whereas protease inhibitors include tissue inhibitors of matrix metalloproteinases-1 (TIMP-1), TIMP-2, and α-2-macroglobulin.^102^ Functional mass spectrometry imaging (fMSI) is a new concept experiment verifying enzyme activity from a new and generalizable method for imaging the biological activity in situ. There are a couple of major drivers for an fMSI approach; the first is to detect the presence of an enzyme by its activity when it is potentially difficult to directly detect, and the second is that the direct detection of an enzyme does not prove its activity. The enzyme needs to be near its substrate and free from inhibitors to function; thus, fMSI can be a powerful approach to show that an enzyme is active in a tissue section. fMSI was initially demonstrated and showed the activity of phospholipase A2 (PLA_2_) in the gland of the brown forest cobra (*Naja subfulva*),^21^ and this activity was then reported as a multiplexed enzyme activity.^103^ In a subsequent investigation conducted by the author’s research team, the efficacy of fMSI in OA progression was validated. Their findings revealed elevated PLA2 activity in both the superficial and deep layers of cartilage in OA-afflicted samples. Notably, the enzyme PLA_2_G_2_A was identified as the predominant catalytic agent by spatial proteomics and immunohistochemistry (IHC), contributing to the spatial progression of OA.^104^ Since fMSI is a broadly applicable approach requiring an enzyme with a substrate that can be detected using MSI, it will play an important part in future OA research where tissue-level enzyme dynamics could be monitored.

### Other essential spatial phenotyping technologies

#### Elemental phenotyping imaging

**Fig. 4:**
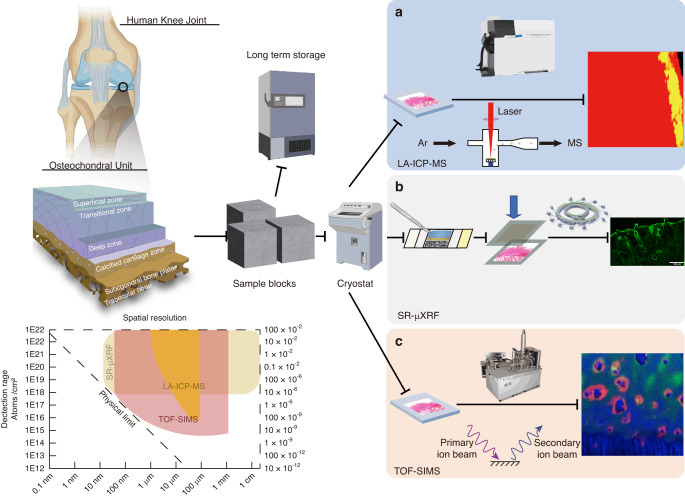
Pipelines of the different spatial phenotyping technologies used in elemental phenotyping imaging. **a** Laser ablation–inductively coupled plasma‒mass spectrometry (LA-ICP‒MS) imaging uses a laser to ablate the tissue, which is then delivered with a carrier gas to the inductively coupled plasma (ICP). The ICP atomizes the sample, and the mass spectrometer analyzer analyses the generated ions. ICP‒MS can analyze sizes as low as 1 μm and has a mass-specific ability to simultaneously monitor several elements. **b** Synchrotron-micro X-ray fluorescence (SR-μXRF) imaging uses synchrotron radiation as an excitation source. An inner shell electron of the atom is struck and ejected by the X-ray. Fluorescence radiation is emitted and measured once an electron from a further outer shell fills the vacant shell site with a spatial resolution of up to 1 μm. This technique enables quantitative measurements that spatially detect elements. **c** Time-of-flight secondary ion mass spectrometry (TOF-SIMS) imaging dislodges chemical species on a material’s surface using a focused, pulsed ion beam. The time of fight of the produced dissociated ions for elemental and some molecular components with spatial resolution as low as 50-100 nm are detected. The spatial resolution depends upon the focused ion beam used, which is contingent on the sample type being analyzed. It has the advantages of a wide detection range, high accuracy and sensitivity. The use of this technique for bone analysis can be challenging due to charge accumulation during analyses. However, new-generation instruments are more effectively mitigating charge buildup. The results are only semiquantitative, and companion XPS analysis can often improve the associated quantitation. This figure was created with BioRender.com

Elemental distribution changes during OA are an integral part of OA progression. Mineral rearrangement and remodeling are deeply correlated with changes in cartilage thickness, calcified cartilage, and subchondral bone layer.^105,106^ Moreover, multiple enzymes are bound to metal ions at their binding site to exert their physiological function. However, discovering the authentic element distribution is difficult, as chemical fixation constantly changes the distribution of the elements.^107^ These studies require fresh frozen tissue where no delocalization or removal of chemical species has occurred. Components such as cations, anions and small molecules in a water-soluble environment can be readily removed by washing (Fig. 4). A detailed comparison of the elemental mapping is listed in Table 3.

**Table 3:** Comparison between the different advanced spatial phenotyping techniques for elemental phenotyping imaging

Imaging technique	Representative technique	Working Principle	Resolution	Advantages	Disadvantages	Metabolite detected	Application in OA
Elemental phenotyping imaging	LA-ICP‒MS	Use laser ablation to create fine particles. Deliver ablated particles to the ICP‒MS instrument’s secondary excitation source for digestion and ionization. then analyze the plasma torch’s excited ions by mass spectrometry	25 μm	Quantitative measurement that detects elements spatially	Relatively low accuracy and spatial resolution	Ca, P, S, Zn, Na, Mg, K^106,181^	Reflects the spatial expression of elemental distribution in the OA tissue
	SR-μXRF	Utilizing synchrotron radiation as an excitation source. An inner shell electron of the atom is struck and ejected by an X-ray. Fluorescence radiation is emitted and measured once an electron from a further outer shell fills the vacant shell site.	1 μm	Quantitative measurement that detects elements spatially	Need synchrotron expert’s assistance, expensive experiment, and competitive application	Ca, P, S, Zn, Pb, Sr^22^	Reflects the spatial expression of elemental distribution in the OA tissue in high-resolution
	TOF-SIMS	Dislodging chemical species on a material’s surface using a focused, pulsed particle beam detects the time of fight of the produced dissociated ions for elemental detection.	0.1 μm to 50 nm^111^	Wide detection range, high accuracy and sensitivity	Difficult on the bone surface because of charging, semiquantitative	Ca, P, Cl^95^	Reflects the spatial expression of elemental distribution in the OA tissue in high-resolution

*LA-ICP‒MS* Laser ablation‒inductively coupled plasma‒mass spectrometry, *SR-μXRF* Synchrotron radiation micro X-ray fluorescence

Previously, several studies were performed to investigate OA changes using X-ray-based fluorescence, including energy-dispersive X-ray spectroscopy (EDS),^106,108^ X-ray fluorescence (XRF),^109^ and laser ablation inductively coupled plasma‒mass spectrometry (LA-ICP‒MS).^106^ However, these technologies have drawbacks regarding quantification, sensitivity and accuracy. EDS and EBSD are surface-sensitive instruments that detect 10–20 nm of the surface. However, the samples’ surface topography affects the data, and the data cannot be quantified using the current EDS system. XRF and LA-CP-MS can be used to quantify the data generated by the resin osteochondral sample.^106^ However, the imaging resolution is relatively low (~25 μm), causing a more challenging detection of the subcellular and osteochondral interfaces. A long processing time also causes difficulty in imaging.

However, with the optimization of sample preparation, the author’s research group successfully developed a 3-layer sandwich that could be used for fresh-frozen elemental detection in synchrotron radiation micro X-ray fluorescence (SR-μXRF) imaging.^22^ This instrument has ultrahigh resolution (~1 μm) at a relatively fast speed and enables applications due to its nondestructive characteristics. We also found a distinct elemental pattern between normal and OA conditions. However, these technologies only have a higher sensitivity to metal atoms heavier than silicon.

Time-of-flight secondary ion mass spectrometry (TOF-SIMS) is a powerful surface analysis technique that directs focused ion beams on surfaces for the generation of secondary ions that are extracted into the TOF analyzer. TOF-SIMS is very effective for mapping elements and some small molecules. Moreover, it has broad detection spectra, from hydrogen to uranium. The interpretation of small molecule analysis can be complicated, as the secondary ions are often fragments of other ions, even though the molecular ions of some species can be observed. TOF-SIMS is relatively nondestructive, allowing for follow-up analyses to be performed on the same physical sample, and it is also relatively fast and can deliver a spatial resolution as low as 70 nm depending upon the focused ion beam. For molecular studies, TOF-SIMS instruments are now more routinely able to provide MSMS imaging analysis, which can be very important to differentiate isomeric or isobaric species observed during MS-TOF-SIMS experiments. TOF-SIMS imaging has been used in the OA field. Cillero-Pastor et al. first used TOF-SIMS to detect elemental and functional group changes during OA progression and found Ca deposits in the ECM and low chlorine signals in OA samples compared with those of healthy samples.^95^ Jung et al. used TOF-SIMS and detected elevated calcium (Ca) and colocalization with phosphate (Pi) in early-stage OA articular cartilage. The Ca-Pi complex enhanced MMP-3 and MMP-13 synthesis in hypertrophic chondrocytes, which indicated that the Ca-Pi complex is a potential mediator in OA progression.^110^ To date, new nanoSIMS has reduced the spatial resolution to 50 nm, thus enabling elemental or isotopic distribution at the nanoscale.^111^ In summary, the above instrument can dynamically and accurately trace the elemental changes.

#### Mechanical phenotyping

**Fig. 5:**
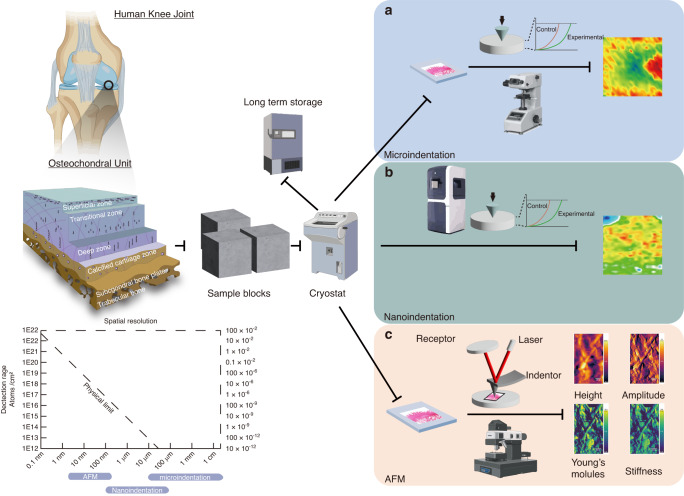
Pipelines of the different spatial phenotyping technologies used in elemental phenotyping imaging. **a** Microindentation enables basic elemental mapping using a microindenter. It is easy to use but has relatively low sensitivity and spatial resolution. **b** Nanoindentation enables an improved indentation function with a smaller gap between indentation points and can reach a spatial resolution of 100 μm. However, it has a lower imaging area compared with microindentation. **c** Atomic force microscopy (AFM) uses a cantilever as a nanoindenter with a spatial resolution of up to 1–5 nm. It enables mechanical testing at the molecular level. However, it requires a steep learning curve with a narrow window for imaging. This figure was created with BioRender.com

OA was once believed to be a “wear and tear” disease. Currently, it is recognized as a disease involving the entire joint, is strongly associated with or induced by movement and physical pressures,^112,113^ and characterized by an imbalance of the joint mechanical loading and biomechanical properties. Proper mechanical loading is essential for joint homeostasis, whereas excessive weight bearing and joint loading increase the risk of OA occurrence.^113^ Therefore, a thorough understanding of the mechanical phenotyping of the joint will aid in the understanding of the OA etiology (Fig. 5).

Currently, the most common method of mechanical testing is Instron, which produces the stress‒strain curve, and based on that, Young’s modulus and other mechanical properties can be measured. However, due to its extensive innate design, this widely used method does not provide detailed mechanical mapping. To address this situation, researchers applied microindentation, nanoindentation, and AFM to map the mechanical changes of OA in different stages. Microindentation was the first instrument to map the osteochondral junction using OA progression. Previous research has demonstrated that the osteochondral junction loses flexibility as OA advances, independent of stratigraphic location.^114–116^ For the early detection of OA, a fiberoptic microindentation approach with a sensor using decreased dimensions (0.125 mm in diameter and 27 mm in length) was utilized,^117^ and a decrease in cartilage rigidity on the tibia plateau of OA patients was found. Hartmann et al. further compared fiber-based microindentation with atomic force microscopy-based nanoindentation and found that the microindenter provided a more accurate and precise assessment of cartilage degeneration than the OARSI histological grading system. However, this technique was less sensitive than AFM at identifying the mechanical changes, demonstrating the future application of fiberoptic microindentation uses through arthroscopy.^118^

Atomic force microscopy (AFM) has always been used to determine molecular topography. In recent decades, researchers have developed its capacity to measure mechanical properties at the subcellular level, leading to a great advancement in mechanobiology. The basic working principle of AFM is to use the artificial tip to attach the sample’s surface and test the retraction force of the sample, thus producing the stress‒strain curve and resolving to the sample’s elastic property (Young’s modulus). Stolz et al. introduced this indentation-type atomic force microscopy to measure human and mouse cartilage samples suffering from aging and OA.^23^ However, only grades 1 and 2 OA were detectable with the AFM and not with micrometer indentation (graded by the Outerbridge scale system). This technique has been widely used for treatment efficacy testing and pathophysiology.^119–121^ In summary, a correlation application between fluorescence microscopy and AFM provides more possibilities for future applications.^122^

## Current challenges for the successful translation of spatial imaging techniques

### Technological evolution and complexities in spatial imaging

Successfully implementing spatial imaging requires achieving subcellular resolution and broader detection ranges. Three primary substance identification methods are widely utilized in clinical samples: barcoded oligos,^123^ aggregated elements, and molecular weights extracted from a mass spectrometry database. For OA studies, MSI is gradually gaining recognition. It incorporates three ionization methods: SIMS (providing high spatial resolution with a limited mass range), MALDI (providing medium spatial resolution and an extensive mass range, with higher resolution in transmission mode), and DESI (nondestructive with lower resolution and a medium mass range). Recent advancements in detectors, including Orbitraps, Fourier transform ion cyclotron resonance (FT/ICR), and TOF reflectors, provide ultrahigh mass resolution (>1 000 000)^124^ that can distinguish isotopes in complex substances. Ion mobility (IM) further refines the reliability of peak assignment, particularly for lipids and metabolites. Additionally, ozone-induced dissociation has emerged in lipid detection, leveraging gas-phase interactions to enhance the discernment of lipid distribution in OA tissue.^125,126^ These evolving technologies have the potential to revolutionize our understanding of OA by providing precise molecular and spatial data, thereby promising more accurate diagnoses and targeted treatments.

### Optimizing sample preparation methods for deep spatial phenotyping

Sample preparation for bone and cartilage tissue poses a challenge. Formalin-fixed, paraffin-embedded, or resin samples are commonly used in research. However, these methods induce alterations to the tissue ultrastructure through crosslinking, chemical fixation, and washing. New technologies provide tools for spatial analysis of this tissue. Fresh-frozen sections now allow advanced imaging for the spatial characterization of elements, metabolites, proteins, RNA, and DNA.^73^ Multiple commercially available tape-assisted systems are designed to preserve the integrity of cartilage and bone.^127^ This innovative sample preparation approach that preserves the original status of bone and cartilage tissue is needed for spatial-level imaging techniques for accurate representation of the disease mechanisms.

### Artificial Intelligence and deep learning for the segmentation, clustering, and annotation of tissue

In the imaging domain, vast data necessitate advanced deep learning and AI applications in biomedical areas such as medical image processing and prediction. Technological advancements have led to multiple image clustering methods based on characteristic peaks. One approach utilizes nanoproteomics at each data point, achieving up to 100 μm resolution.^128^ Another merges precursor signals from data points with LC-MS/MS datasets, notably the High-resolution Informatics Toolbox in MALDI-MSI Proteomics (HiT-MaP).^24^ An analytical algorithm evaluates the peptide signatures by contrasting actual and projected isotopic distributions. This approach has been effectively applied to the imaging and analysis of corneal tissue. The software has also been invented for automated lipid imaging detection, including Alex,^129^ LipostarMSI,^130^ MassPix,^131^, and common alkali metal adducts, matrix adducts, and isotopes, including Mass2adduct.^132^ Common annotation platforms, including pySM (METASPACE), have also been developed for lipid and metabolite annotation with high mass accuracy data.^25^ However, this method relies more on predicting peaks and combining the MS/MS dataset. Artificial intelligence and deep learning algorithms have become increasingly prevalent in the standard categorization of OA by combining data such as histopathological staining,^133^ X-rays,^134^ MRI,^135^, and blood tests.^136^ However, hyperspectral and molecular imaging as a potential application area for these technologies has not been explored and potentially provides a significant opportunity for innovation and research.

### The database enables the pooling, integration, and analysis of the data

Currently, no database has been built directly for OA, although multiple proteomic studies have been performed regarding cartilage,^137^ bone^138^, and synovial fluid.^139^ However, multiple human databases have been built for reference in MSI, which covers proteins (UniProt,^140^ MSiMass list database,^141^ and MaTisse^142^), lipids (LipidMaps,^143^ LipidBank^144,145^), metabolomics (CoreMetabolome, HMDB,^146,147^ ECMDB,^148^ KEGG,^149^ and METLIN^150^), pharmaceutics (DrugBankand) and N-glycan (NGlycDB). Commercial databases, such as SCiLS Lab, also allow a certain degree of annotation of the mass to be used in the OA field. However, all the databases only show the mass acquisition of the substance, and the exact composition, such as isomer determination, still needs to be determined by MS/MS analysis.

## Therapeutic potential of spatial imaging in OA research

**Fig. 6:**
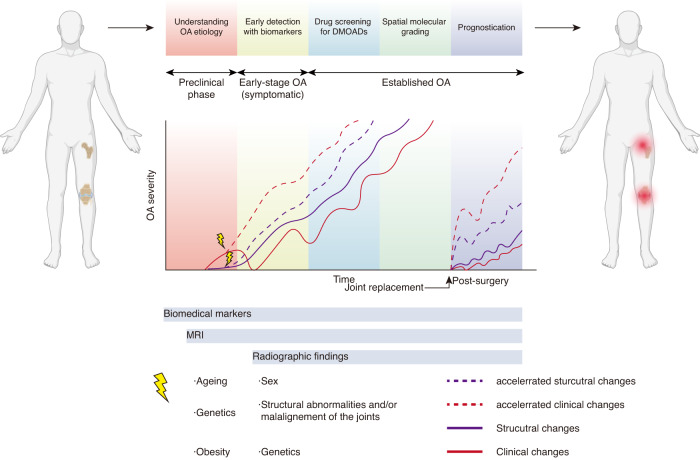
Landscape of clinical application in all stages of OA with deep spatial phenotyping techniques. Deep spatial phenotyping techniques can be used for understanding OA etiology, early detection with integrated biomarkers, drug screening for disease-modifying drugs for OA (DMOADs), spatial molecular grading, and prognostication of OA. This figure was created with BioRender.com

Applying spatial imaging techniques can transform our comprehension of OA, providing pivotal insights into disease mechanisms, optimizing risk assessment and patient stratification, and elevating the accuracy of prognosis and diagnosis (Fig. 6).

### Understanding OA disease mechanisms

Spatial imaging techniques can show the intricate mechanisms behind OA, and they allow researchers to visualize the spatial distribution of molecular mechanisms within joint tissues. Techniques such as spatial transcriptomics, spatial proteomics, and spatial metabolomics show molecular signatures, clarifying inflammation, metabolic shifts, genetic influences, and mechanical stresses. This spatial insight aid in the better understanding of OA as a multifaceted inflammatory disease that affects the entire joint. The previous oversimplification of OA classification into primary and secondary forms is being challenged, facilitating a deeper comprehension of OA’s underlying mechanisms and risk factors, particularly different OA phenotypes and endotypes.^151^

### Risk assessment and patient stratification

Spatial imaging may enhance OA risk assessment and patient stratification. By analyzing spatial patterns of biomarkers and molecular signatures, we can identify high-risk patients and tailor treatments based on their unique molecular profiles, including comorbidities such as cardiovascular disease, high BMI,^152^ high cholesterol,^153^ T2DM,^154^ and structural abnormalities. For example, spatial molecular profiling reveals differences in lipid and protein profiles in patients with comorbid conditions such as T2DM.^97^ Subclassifying patients based on these signatures may aid in personalized treatment strategies. Bridging the gap between spatial molecular data and conventional imaging can revolutionize our understanding of OA and patient stratification. X-ray and MRI techniques are currently used for clinical diagnosis but provide only semiquantitative assessments with limited sensitivity, particularly in early OA. However, these methods fall short in the effective analysis of patient stratification. OA represents a diverse range of clinical phenotypes and molecular endotypes, encompassing inflammatory, metabolic, mechanical, genetic, and synovial aspects. Due to this complexity, innovative technologies are essential for accurate diagnosis. In previous research, we highlighted the effectiveness of near-infrared (NIR) imaging in spatially detecting OA-related changes in both cartilage and subchondral bone. The integration of hyperspectral imaging with arthroscopy has promise for real-time diagnoses and a comprehensive understanding of tissue composition.^155,156^ Additionally, infrared spectroscopy (IR) imaging enables the detection of specific chemical bonds in tissues, providing insights into molecular alterations in joint diseases.^28^ Raman spectroscopy imaging provides detailed information about the molecular makeup of joint tissues, including proteins and lipids, enhancing our understanding of molecular changes in OA.^157^ Mass spectrometry imaging enables the precise measurement of molecular masses in joint tissues, aiding biomarker identification.^158^ Fluorescence spectroscopy is another method for studying specific biomarkers within joint tissues,^159^ contributing to our understanding of their distribution and composition. Despite their potential, these techniques are not widely employed in clinical practice for OA. Instead, they are commonly used in laboratory settings to study the molecular composition and characteristics of joint tissue. Therefore, translating these techniques into clinical practice requires further research and development efforts and collaboration between academia, health care providers, and industry.

### Prognosis and diagnosis

A recent study by Barré et al.‘s systematic analysis emphasized that a single biomarker does not determine OA.^28^ Therefore, spatial imaging has the potential to significantly improve the prognosis and diagnosis of OA by providing a detailed spatial distribution of multiple marker changes within the joint. This improvement allows clinicians to make more accurate prognoses. Spatial imaging combines various methods, including NIR,^28^ FTIR,^40^ and Raman spectroscopy,^55^ with currently feasible arthroscopy, contributing to early detection and diagnosis. The integration of spatial phenotyping, coupled with mapping relationships between tissue changes and circulatory disease indicators in blood,^160,161^ urine,^161^ and saliva^162,163^ using advanced machine learning and bioinformatics tools, has the potential to enhance early detection and intervention strategies for OA.

### Precision treatment

Currently, the application of spatially resolved techniques in evaluating potential drugs for OA is not widespread. However, this technique has already found extensive application in cancer and renal disorders.^164,165^ A pioneering study by Barré et al. examined this approach and explored the effect and distribution of triamcinolone acetonide on human OA cartilage. Their research successfully illustrated the penetration depth of this corticosteroid drug in human OA cartilage using MSI.^166,167^ The spatial resolution of these techniques enables targeted treatment delivery, minimizing the collateral damage to healthy tissues. Furthermore, MSI can trace drugs on a spatial scale, enabling the validation and reliability of outcomes. This facilitates more controlled drug release and the tracing of various metabolites.

#### Future directions

The advancement in deep spatial phenotyping in OA heavily relies on developing commercially available instruments. The future direction of deep spatial phenotyping relies on more accurate annotation and higher instrument accuracy. More subcellular experiments in situ can aid in the determination of the underlying mechanism of OA progression and help find a potential treatment. Currently, the transmissive mode of MALDI-2 MSI allows for subcellular analysis of the mass with higher resolution (~600 nm) in combination with higher mass accuracy.^168^ Cost-effectiveness is another issue that needs to be resolved in future studies. More cost-effective methods or adaptations need to be optimized to accommodate various studies. Moreover, moving from 2D to 3D imaging is another advancement. No study has directly used the 3D animal model in OA. According to Seeley et al., 3D reconstruction is more challenging than two-dimensional imaging because sectioning artifacts such as tears, folds, and deformations become more critical.^169,170^ Another exciting aspect of deep spatial phenotyping in OA research is the possibility of spatiotemporal analysis, which involves time as a second variable. To date, no studies have investigated this in the context of OA, but it has the potential to provide valuable insights into the dynamic changes in mass that occur during the progression, intervention, and endpoint of OA. Therefore, spatiotemporal analysis could be particularly useful for drug screening. Some prototype machines already have a real-time display function that enables short-term tracing, while longitudinal tracking can be used in the future to dynamically track mass changes over time.

Finally, while multiple instruments have taken advantage of various aspects of deep spatial phenotyping, a comprehensive solution that enables a systematic top-to-bottom analysis has yet to be developed. Future studies need to focus on nondestructive sequential annotation, which enables real spatial multiomics studies, helps advance our understanding of OA, and supports the discovery of new therapeutic strategies.^171^

## Conclusions

In conclusion, integrating state-of-the-art imaging techniques with artificial intelligence and deep learning has provided a comprehensive approach to investigating the spatial phenotyping of osteochondral samples in OA research. This multidisciplinary approach has the potential to significantly enhance our understanding of this debilitating disease, which affects millions of individuals worldwide. A deeper understanding of the underlying mechanisms of OA can provide valuable insights into developing effective disease-modifying osteoarthritis drugs (DMOADs) for its treatment and, ultimately, improve the quality of life for patients affected by this condition.
